# Ecological niche divergence between extant and glacial land snail populations explained

**DOI:** 10.1038/s41598-021-04645-2

**Published:** 2022-01-17

**Authors:** Michal Horsák, Veronika Horsáková, Jan Divíšek, Jeffrey C. Nekola

**Affiliations:** Department of Botany and Zoology, Faculty of Science, Masaryk University, Kotlářská 2, 61137 Brno, Czech Republic

**Keywords:** Palaeoecology, Palaeoecology

## Abstract

The presence of Last Glacial Maximum (LGM) biotic communities without modern counterparts is well known. It is particularly evident in central European fossil LGM land snails whose assemblages represent an odd mix of species that are currently limited to either xeric or wetland habitats. Here we document a genetically verified discovery of the modern calcareous wetland species *Pupilla alpicola* on Iceland, where it is limited to dry grasslands. This species also represents a common European LGM fossil, and its new records from Iceland help explain puzzling shifts of some glacial land snails of xeric grassland habitats to open wetlands today. Similarities between the climates of modern Iceland and LGM Eurasia suggest that this species did not become limited to wetlands in continental Europe until after the Late Pleistocene–Holocene climate transition. These results are a strong reminder that assumptions of ecological uniformity must be questioned and that the quality and robustness of palaeoecological reconstructions is dependent upon adequate knowledge of the full autecological range of species over time.

## Introduction

It is not uncommon for full-glacial fossil assemblages to lack modern biological analogues. For instance, fossil assemblages from the European Last Glacial Maximum (LGM) periglacial zone represent a unique mixture of species that currently occupy divergent habitats and/or regions^1,2^. Central North American LGM mammal and pollen records document the existence of an open spruce parkland biome that contained species now characteristic of tundra, taiga, eastern forest, and prairie^3,4^. Multiple mechanisms have been suggested to underlie these patterns, including non-homologous regional climates^3,5,6^, microclimate refugia^7,8^, individualistic species responses to environmental gradients^9^, and varying dispersal abilities^10^.

However, because fossil taxa exhibiting diverse contemporary affinities are commonly found within single deposits^3,11–13^, at least some of the responsible mechanisms must operate at local (or smaller) scales. One possible explanation is fossil material being sourced from a diverse array of adjacent sites. Another is spatio-temporal variation in realized niches. While most palaeoecological reconstructions—based on Lyell’s uniformitarian principle^14^—assume that they remain virtually unchanged over time, altered environmental conditions and biotic interactions make this conjecture questionable^15^.

European full glacial fossil land snail assemblages frequently represent a heterogeneous mix of species now characteristic of either xeric upland or hydric wetland sites^16,17^. Perhaps the best example is *Pupilla alpicola*, which is among the most frequent glacial fossils of European loess deposits^16,18,19^. While modern European and Siberian populations are closely tied to wetlands—mostly calcareous fens^17,20^—its glacial loess fossils typically co-occur with xerophilic species such as *Pupilla sterrii* and *Helicopsis striata*^16,19^. In addition, multiple modern *P. sterrii* populations possess *P. alpicola* mtDNA^21^, in spite of the fact that these populations are all separated by more than tens of kilometres and/or hundreds of meters of elevation^22^. It thus seems likely that the co-occurrence of these two species in fossil deposits is in fact caused by their sharing of the same microsites at some time in the past, rather than by taphonomic consolidation.

Here we use newly discovered populations of *P. alpicola* from Iceland—at least 1500 km from the nearest known continental European sites—to highlight the variability in the species realized niche space. These new records help explain apparent incongruence between site-scale ecological affinities of some glacial land snail fossils and their modern counterparts.

## Materials and methods

### Taxonomy and modern range

*Pupilla alpicola* was recently thought to have its modern range limited to the western Alps east through the Carpathians to Slovakia and Romania^23^. Subsequently, its range was expanded east into the Altai of southern Siberia^17^ and north into Scandinavia^21^. The latter populations had previously been reported as *Pupilla pratensis*^24^ even though DNA data indicate this taxon to be a junior synonym of *P. alpicola*^21^. All modern Iceland populations were previously reported as *P. muscorum*^23,25^ based on an incorrect understanding of its distinguishing shell features^21^.

### Field sampling

In total, 47 modern land snail assemblages were collected in 2021 in Iceland across all suitable habitats, mainly along the coast. These included also several alkaline fens. Some general areas were targeted for sampling due to prior reports of disjunct European elements^25^. The snail fauna of each site was assessed via direct hand picking, dry or wet sieving^17^. All shells were sorted and identified to the species level based on Nekola et al.^21^, Horsák et al.^22^ and Kerney et al.^25^. The latitude/longitude coordinates of each site were recorded via a hand-held GPS device with a resolution of 10 m. Additionally, the general ecological conditions from each site were recorded.

### Identification validation

To validate our identification of Icelandic *Pupilla* as *P. alpicola* rather than *P. muscorum*, phylogenetic analysis was performed on two populations (Table 1). We considered the same two mtDNA (COI, CytB) and nDNA (ITS1 + ITS2) regions of Nekola et al.^21^, and used the same protocols described therein. Sequence traces were assembled using Geneious v. 8.0.2 (Biomatter Ltd.) and uploaded into GenBank (Table 1). Sequences were concatenated into separate mtDNA (COI + CytB) and nDNA (ITS1 + ITS2) constructs and aligned to five European *P. alpicola*, three *P. muscorum*, three *P. triplicata*, and one *P. sterrii* analyzed by Nekola et al.^21^. Phylogenies and associated support values are based on methods presented in Horsáková et al.^26^ and use four fundamentally different methods to ensure robust support [Neighbor-joining (NJ), Maximum parsimony (MP), Maximum likelihood (ML) and Bayesian inference (BI)]. BI trees were used to illustrate the phylogeny.

**Table 1:** Locations of new *Pupilla alpicola* records on Iceland and GenBank accession numbers of two specimens used in phylogenetic analyses.

Site	Habitat	Lat (°N)	Lon (°E)	Sample code
Búðir	Isolated tussocks in meadow tundra on the top of lava outcrops	64.8215	− 23.3865	IS04
Krossanesborgir	Steppe-tundra on dry sandy drift	65.7033	− 18.1386	IS12
Blönduós	Steppe grassland on rocky slope of SW exposition	65.6591	− 20.2679	IS13
Patreksfjörður	Steppe grassland on isolated basalt-column rock of SW exposition	65.5455	− 23.8843	IS21

Sample code	GenBank accession number			
	CytB	COI	ITS1	ITS2
IS04	OL692495	OL684954	OL684952	OL685167
IS12	OL692494	OL684953	OL684951	OL685166

### Climatic modelling

Climate suitability for *P. alpicola* was modelled using 80 genetically validated modern Eurasian occurrences. We retrieved 35 climatic variables for each occurrence at a spatial resolution of 5 arc-minutes from WorldClim v.1.4^27^ and ENVIREM^28^ databases. These data represent biologically relevant variables derived from average monthly climate interpolated among weather stations for the period 1960–1990. Using Maxent software (version 3.4.1;^29^), we generated the characteristic climate envelope for *P. alpicola*. The model was calibrated, using fivefold cross-validation, from the 12 best-performing climatic variables: isothermality (bio3), maximum temperature of the warmest month (bio5), mean temperature of the coldest quarter (bio11), precipitation of the driest month (bio14), precipitation seasonality (bio15), precipitation of the coldest quarter (bio19), Thornthwaite aridity index (an index of the degree of water deficit below water need), potential evapotranspiration (PET) of both the driest and wettest quarters, PET of the coldest quarter, Emberger's pluviothermic quotient (a metric designed to differentiate among Mediterranean type climates) and the count of the number of months with mean temperature greater than 10 °C. The new Iceland records were not used for model parameterization so that it could be determined if observed regional Eurasian climates could accurately predict the Iceland range.

We also mapped the similarity between current climate of Iceland *P. alpicola* sites and LGM climates of the western Palearctic, using MPI-ESM-P global circulation model simulations, which were downloaded from the abovementioned climatic databases. For each variable, we recorded the range of values (± Standard Deviation) in which the modern Icelandic populations of *P. alpicola* currently occur. The final composite map shows climatic similarity as the amount of overlap of these modern climatic ranges mapped within the Eurasian LGM landscape, ranging from dark blue/purple (maximum) to yellow (minimum).

## Results and discussion

**Figure 1:**
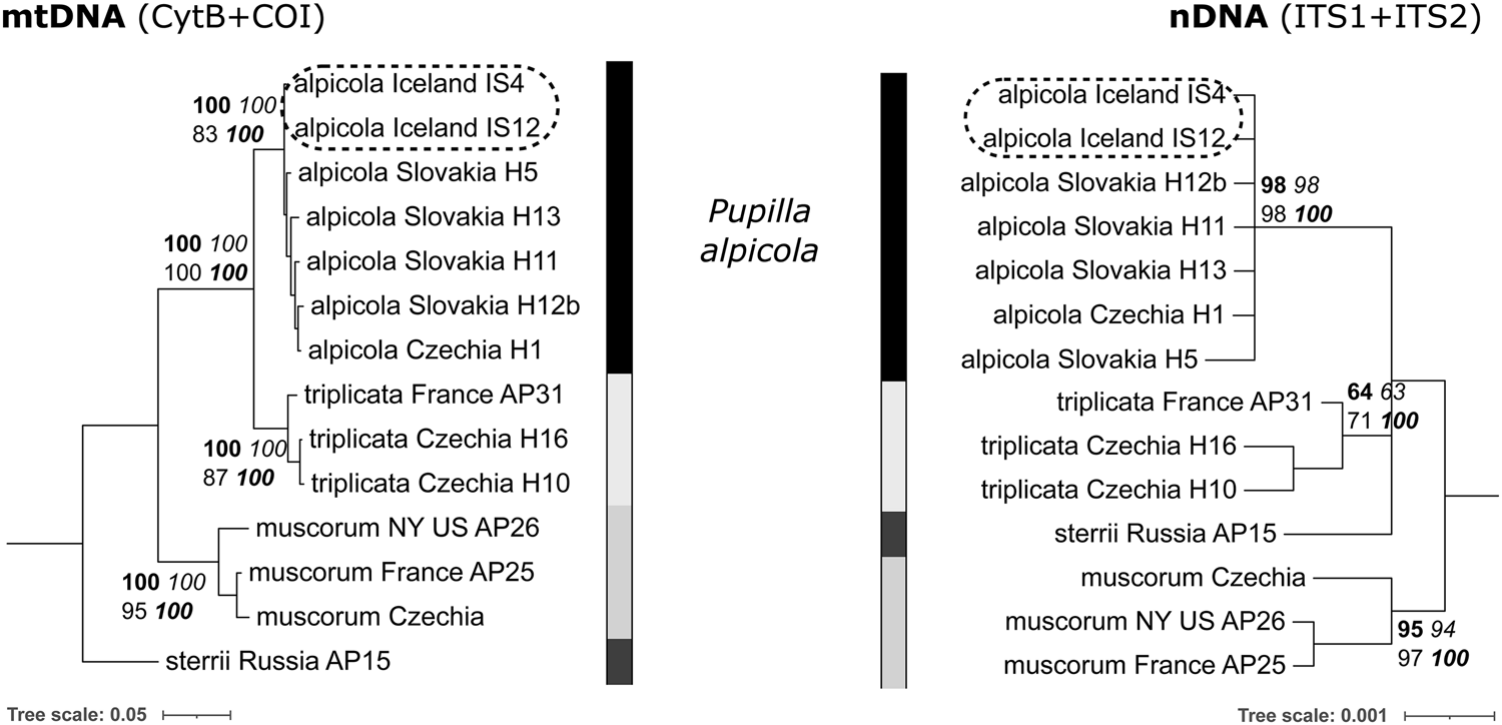
Phylogenetic reconstruction of European *Pupilla* species based on the Bayesian inference of mitochondrial DNA (concatenated CytB + COI, left) and nuclear DNA (concatenated ITS1 + ITS2, right). Support values are shown next to the corresponding nodes as follows: Neighbor joining, upper left, bold; Maximum parsimony, upper right, italic; Maximum likelihood, lower left, normal font; and posterior probabilities for Bayesian inference, lower right, bold italic. Specimens from Iceland (marked by dashed line) clearly fall into the clade of *P. alpicola*^21^.

**Figure 2:**
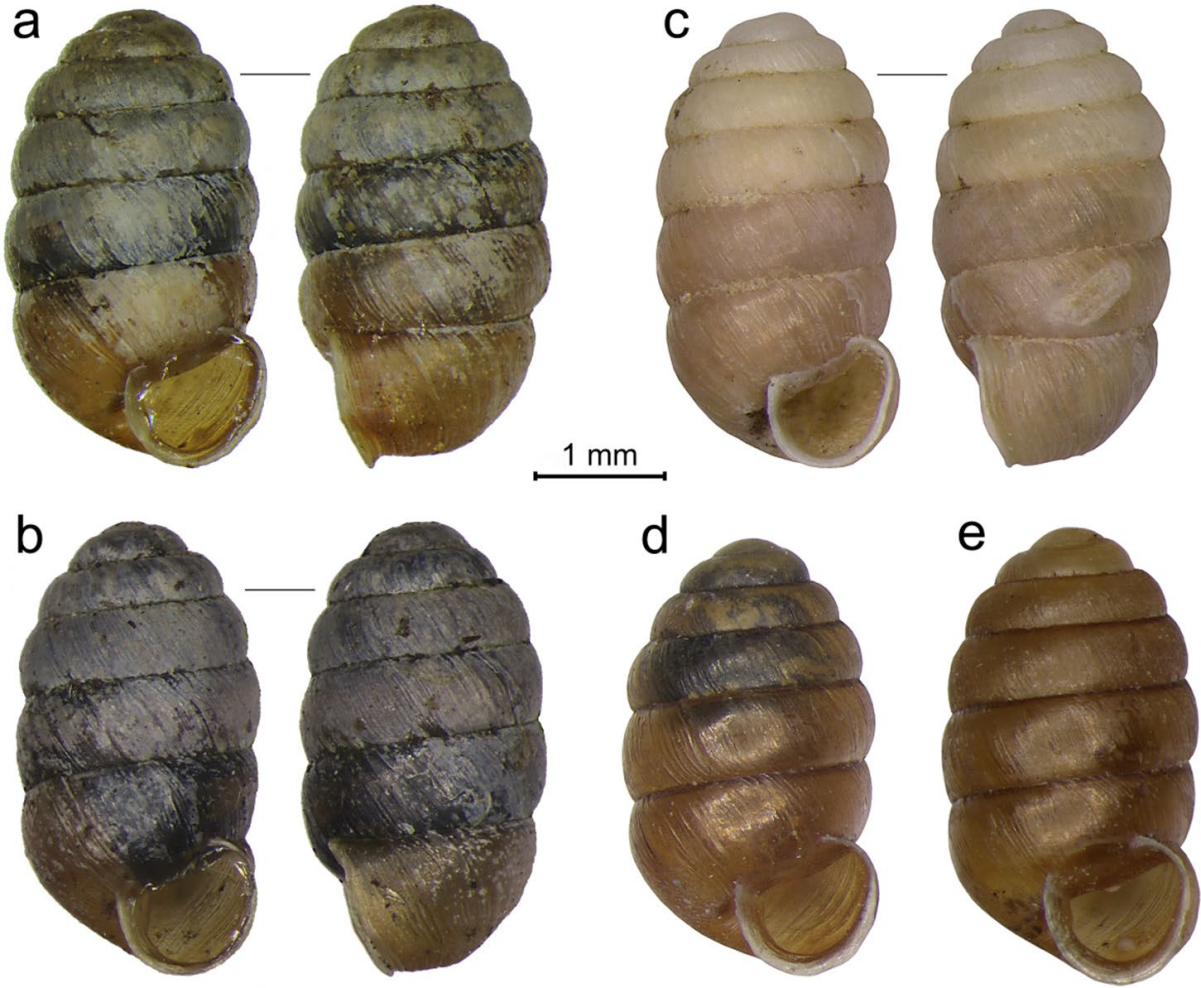
Individuals of Icelandic *Pupilla alpicola* used in genetic analyses (**a**,**b**), a fossil shell from glacial loess deposits (**c**), and individuals from two modern central European populations (**d**,**e**): (**a**) 65.7033° N, 18.1386° W, Krossanesborgir, steppe-tundra vegetation with *Dryas octopetala* on dry sandy drift (Fig. 4a); (**b**) 64.8215° N, 23.3865° W, Búðir, grassy patches in meadow tundra on lava outcrops (Fig. 4b); (**c**) 47.9425° N, 18.6487° E, Čata, LGM loess deposits; (**d**) 49.0686° N, 19.9995° E, Slovakia, Važec, calcareous spring fen (Fig. 1, sample no. H11); (**e**) 49.2340° N, 17.9865° E, Czechia, Pozděchov, alkaline fen meadow (Fig. 1, sample no. H1).

Across all four phylogeny reconstruction methods, the material from Iceland was assigned with very high support to *Pupilla alpicola* and not the other three European *Pupilla* species (Fig. 1). Both mtDNA and nDNA data thus confirm the Icelandic populations to represent *P. alpicola*. Even though previously reported as *P. muscorum*, Iceland shells exhibit regularly dense shell striae, and almost no apertural crest and lamellae (Fig. 2a,b), which distinguish them as *P. alpicola*^21^. These populations have shells resembling, by their narrower cylindrical shape, those from glacial loess deposits (Fig. 2c). In contrast, modern populations of continental European spring fens are characteristic by wider ovate shells (Fig. 2d) and also more strongly calcified apertural barriers in *pratensis* form (Fig. 2e), being mainly associated with non-spring wetlands^30^. This might indicate that shell shape is under ecophenotypic control in this species.

**Figure 3:**
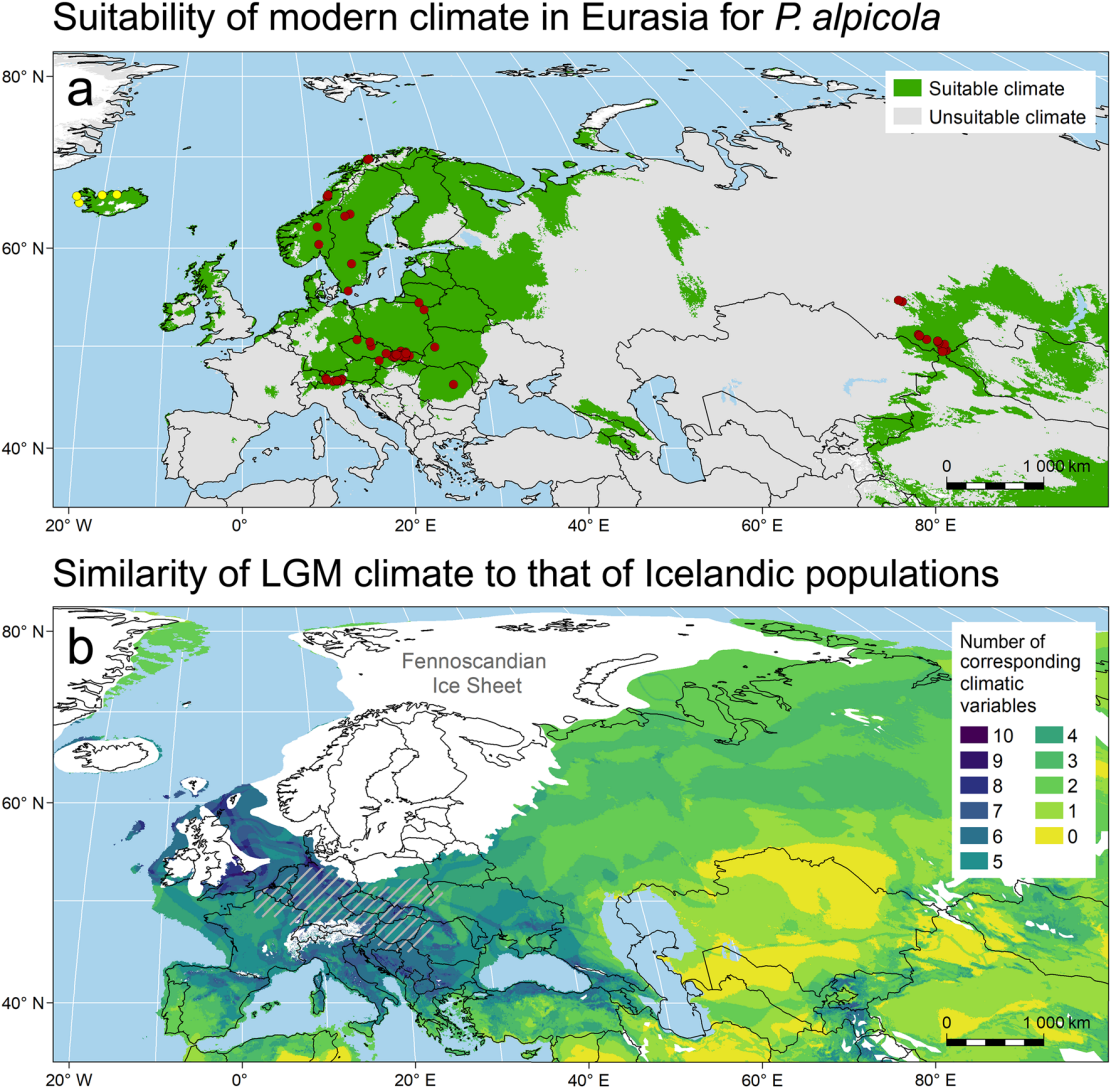
(**a**) Area of appropriate regional climate (marked in green) predicted by Maxent model based on 12 climatic variables gathered for 80 verified modern populations of *Pupilla alpicola* (red dots) across the entire known range. Four new populations from Iceland (yellow dots) were not used in the modelling approach; (**b**) similarity of LGM climate to that of the four modern Icelandic populations. Similarity is expressed as the number of climatic variables whose values are within the range (± SD) recorded for sites where *P. alpicola* is present (yellow dots in **a**). The more variables are overlapping the more similar LGM climate was to the modern climate on Iceland. The hatched area depicts a schematic distribution of fossil loess records of *P. alpicola* in Europe^16,18,31^. The extent of LGM ice sheets was adopted from Ehlers et al.^37^.

Climate niche modelling based on validated modern Eurasian sites indicates that the regional climate for NW Iceland is appropriate for *P. alpicola* (Fig. 3a). Additionally, the area of highest regional climatic overlap between modern Icelandic *P. alpicola* sites and LGM Europe (Fig. 3b) corresponds with the area of known fossil records from loess deposits^16,18,31^. The modern Iceland climate may thus represent a reasonable analogy to LGM climates of central Europe^32^. These analyses also suggest that in the LGM the species should have been present south of the Alps and in the ice free corridor between the British Isles and Scandinavia. The fact that no fossils are known from these regions is likely due to submergence of deposits under the Adriatic and North seas as well as the lack of appropriate LGM sediments and verified data from Mediterranean Europe.

**Figure 4:**
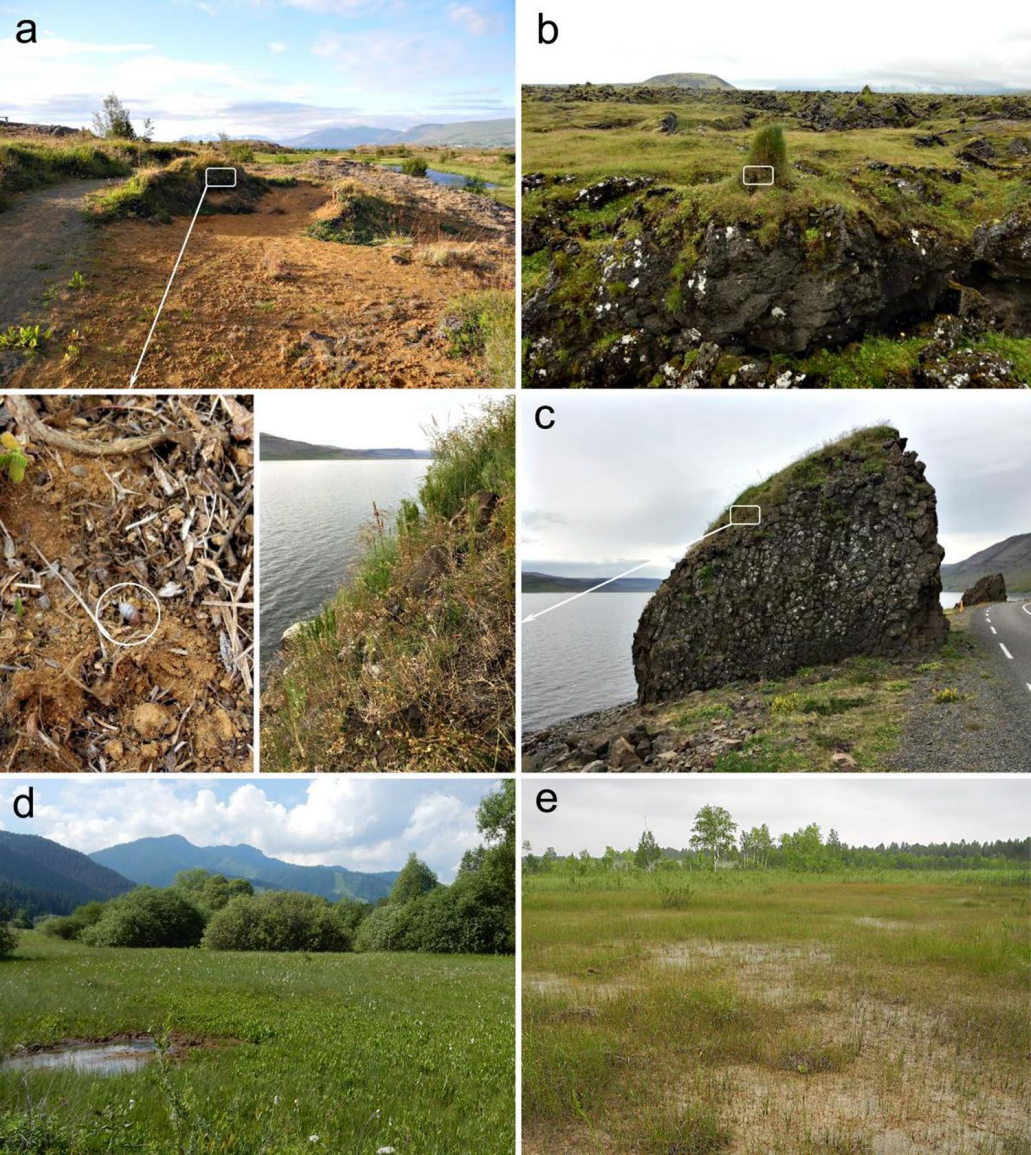
Habitats of modern *Pupilla alpicola* populations from the northwestern coast of Iceland (**a**–**c**) and continental Eurasia (**d**,**e**): (**a**) Krossanesborgir, steppe-tundra vegetation with *Dryas octopetala* on dry sandy drift; (**b**) Búðir, isolated tussocks in meadow tundra on the top of lava outcrops; (**c**) Patreksfjörður, steppe grassland developed on an isolated basalt-column rock of SW exposition; (**d**) calcareous fen in the Western Carpathians (Liptov region, Slovakia); (**e**) calcareous fen in southern Siberia (Kemerovo region, Russia). White frames indicate patches with an abundant occurrence of live individuals with one of them marked by a white circle; arrows point to a zoom-in view on these patches.

The habitat characteristics for the four Iceland sites were remarkably different from all prior known *P. alpicola* sites: rather than occurring in base-rich wetlands, populations were restricted to grass litter accumulations in dry to mesic grasslands on sand dunes, lava or other rocky outcrops (Fig. 4a–c). Soils were typical for steppe habitats (Fig. 4a). Unlike Eurasian fen sites (Fig. 4d,e), Iceland populations also completely avoided areas supporting bryophytes. The Iceland populations also differed in their strict avoidance of microhabitats with tundra-like vegetation. While high accumulations of live individuals were found in thick tussocks under a layer of dead grass litter (Fig. 4b), they were not present nearby in mesic meadows or calcareous fens. Because the occupied habitat corresponds closely to typical occurrences for *P. muscorum*, it is not surprising that these populations had previously been confused with that species^25^.

Although the species is currently very rare on Iceland, the relatively long distance between sites along the northwest Icelandic coast suggests that the current distribution might be a remnant of a more widespread past occurrence. Given the modern day character of the Icelandic landscape, we speculate that population loss has been driven by sheep overgrazing^33^ and concomitant erosion at the landscape level^34^. In favour of this hypothesis, all extant sites were either inaccessible to sheep (Fig. 4c) or occurred within protected conservation reserves (Fig. 4a). It is thus possible that the observed realized niche for *P. alpicola* in Iceland has been anthropogenically limited to only places which exclude sheep and other free-ranging livestock.

Despite the rarity, these records extend the known realized niche for *P. alpicola* into an entirely new suite of dry-mesic to xeric sites that are utterly unconnected with surface exposure of groundwater^20^. The factors common to all known *P. alpicola* sites appear as for their LGM loess environment^16^ to be: (1) high calcium availability, either from base rich bedrock/soil (limestone, amphibolite, basaltic lava, glacial till) or highly calcium-rich groundwater (spring fens/fen meadows) and, (2) low maximum summer temperatures. At the southern end of its range these conditions are usually provided by thermal buffering via groundwater^35^. In Siberia or coastal Norway, where maximum summer temperatures are lower, populations extend into wetland/mesic sites not associated with upwelling groundwater. Some coastal populations in northern Norway occur on mesic, calcareous, rocky slopes^24^, and thus represent an intermediate habitat between Eurasian fens and Icelandic steppe grassland sites.

The Icelandic environment combines young volcanic bedrock with sufficient calcium, and a hyper-maritime climate without extreme summer temperature peaks. These conditions apparently have allowed *P. alpicola* to expand its realized niche into xeric grassland sites. Because high calcium availability (from loess) and lack of summer temperature extremes (from nearness to the continental and Alpine ice sheets) also likely characterized the European LGM environment, it is clear that the LGM habitats for this species in central Europe may have also included xeric grasslands, and not exclusively “wet loess” sites as was previously presumed. It also elegantly explains its co-occurrence with typically xeric species, and the presence of mitochondrial introgression with the steppe *P. sterrii*.

It seems likely a similar mechanism is responsible for the LGM shift to xeric habitats in *Columella columella*^16,36^, another characteristic full-glacial land snail species^18,31^. Some modern *C. columella* populations in the high Alps and Carpathians are known to inhabit mesic tundra as well as fens^17,25^. However, because this species was never able to colonize Iceland, there is no way to determine if xeric sites in that landscape could support its occurrence, or to empirically show that its realized niche is able to expand in this way.

The presence of unique land snail assemblages (and likely other sedentary small taxa like many soil invertebrates, bryophytes, small vascular plants) in the central European LGM thus may not require non-homologous regional climates, microclimate refugia or differential species sorting rules. Rather, they may be explained by simple shifts of realized niches in response to different regional climate and environmental conditions. This work thus should serve as a cautious reminder that constrained adherence to uniformitarianism regarding habitat requirements can limit palaeoenviromental interpretations and reconstructions, as well as species distribution models.

## Data Availability

All data is available in the main text or Table 1.
